# A Gain-of-Function Mutation in the Ca^2+^ Channel ORAI1 Causes Stormorken Syndrome with Tubular Aggregates in Mice

**DOI:** 10.3390/cells13221829

**Published:** 2024-11-06

**Authors:** Laura Pérez-Guàrdia, Emma Lafabrie, Nadège Diedhiou, Coralie Spiegelhalter, Jocelyn Laporte, Johann Böhm

**Affiliations:** Institut de Génétique et de Biologie Moléculaire et Cellulaire (IGBMC), Inserm U1258, CNRS UMR7104, Université de Strasbourg, 67404 Illkirch, France

**Keywords:** myopathy, calcium, Stormorken syndrome, ORAI1, STIM1, SOCE

## Abstract

Store-operated Ca^2+^ entry (SOCE) controls Ca^2+^ homeostasis and mediates multiple Ca^2+^-dependent signaling pathways and cellular processes. It relies on the concerted activity of the reticular Ca^2+^ sensor STIM1 and the plasma membrane Ca^2+^ channel ORAI1. STIM1 and ORAI1 gain-of-function (GoF) mutations induce SOCE overactivity and excessive Ca^2+^ influx, leading to tubular aggregate myopathy (TAM) and Stormorken syndrome (STRMK), two overlapping disorders characterized by muscle weakness and a variable occurrence of multi-systemic anomalies affecting spleen, skin, and platelets. To date, different STIM1 mouse models exist, but only a single ORAI1 mouse model with muscle-specific TAM/STRMK phenotype has been described, precluding a comparative analysis of the physiopathology in all affected tissues. Here, we generated and characterized mice harboring a prevalent ORAI1 TAM/STRMK mutation and we provide phenotypic, physiological, biochemical, and functional data. Examination of *Orai1^V109M/+^* mice revealed smaller size, spleen enlargement, reduced muscle force, and decreased platelet numbers. Morphological analyses of muscle sections evidenced the presence of tubular aggregates, the histopathological hallmark on biopsies from TAM/STRMK patients absent in all reported STIM1 models. Overall, *Orai1^V109M/+^* mice reliably recapitulate the human disorder and highlight the primary physiological defects caused by ORAI1 gain-of-function mutations. They also provide the possibility to investigate the formation of tubular aggregates and to develop a common therapy for different TAM/STRMK forms.

## 1. Introduction

Tubular aggregate myopathy (TAM, OMIM #160565 and #615883) is characterized by progressive muscle weakness associated with the occurrence of densely packed membrane tubules in myofibers [1]. Age of onset and disease severity are heterogeneous and depend on the implicated gene and the position of the mutation. To date, four different TAM genes have been described, *CASQ1* [2,3], *RYR1* [4], *STIM1* [5], and *ORAI1* [6,7], all encoding major regulators of Ca^2+^ homeostasis. Patients with pathogenic variants in the muscle-specific reticular Ca^2+^ buffer calsequestrin (CASQ1) or the muscle-specific reticular Ca^2+^ channel RyR1 form the mild end of the clinical TAM spectrum and typically manifest moderate adult-onset muscle weakness and exercise-induced muscle pain and stiffness [2,3,4,8]. In contrast, mutations in the ubiquitous reticular Ca^2+^ sensor STIM1 or the ubiquitous plasma membrane Ca^2+^ channel ORAI1 give rise to a multi-systemic phenotype involving muscle weakness in combination with a variable degree of additional signs such as miosis, ichthyosis, short stature, thrombocytopenia, and hyposplenism [7,9,10,11]. The full clinical picture constitutes the diagnosis of Stormorken syndrome (STRMK, OMIM #185070) [12].

**Figure 1:**
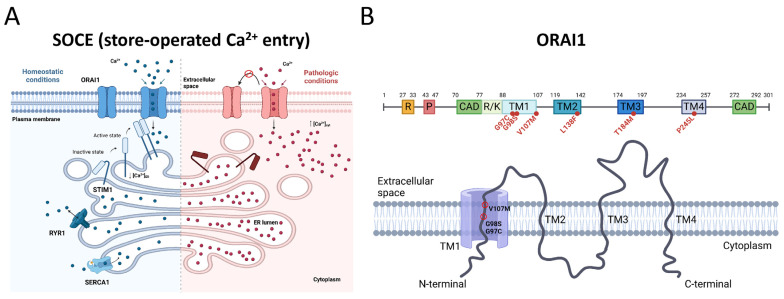
SOCE (store-operated Ca^2+^ entry) and ORAI1 domains. (**A**) In normal conditions, reticular Ca^2+^ store depletion induces STIM1 unfolding, oligomerization, and interaction with ORAI1 to trigger extracellular Ca^2+^ entry (**left**). In TAM/STRMK, ORAI1 gain-of-function (GoF) mutations generate a leaky channel and induce excessive Ca^2+^ influx independently of the reticular Ca^2+^ load and STIM1 binding, resulting in elevated Ca^2+^ levels in the cytosol and the reticulum (**right**) [9]. (**B**) Schematic representation of the ORAI1 protein domains with position of the described GoF mutations (**above**). ORAI possesses 4 transmembrane domains (TM1–TM4). G97C, G98S, and V107M affect conserved amino acids in TM1, forming the channel pore (**below**). R = arginine-rich, P = proline-rich, CAD = CRAC channel activating domain; R/K = arginine/lysine-rich.

STIM1 and ORAI1 form the key components of store-operated Ca^2+^ entry (SOCE), an elemental mechanism mediating extracellular Ca^2+^ entry to replete the intracellular Ca^2+^ stores and trigger Ca^2+^-dependent pathways in all cell types [13,14]. Ca^2+^ allosterically regulates enzymes and acts as a second messenger for signal transduction in neuronal transmission, T-cell differentiation, hormone secretion, coagulation, and muscle growth and contraction. Hence, normal physiology relies on the strict regulation of Ca^2+^ entry, storage, and release. Functional investigations in cellular models demonstrated that the TAM/STRMK-related *STIM1* and *ORAI1* mutations involve a gain of function and induce SOCE over-activation or constitutive ORAI1 channel permeability, resulting in excessive Ca^2+^ influx (Figure 1) [2,5,6,7,11,15,16,17,18].

Several mouse models harboring different STIM1 mutations have been described. In compliance with the clinical presentation of TAM/STRMK patients, *Stim1^D84G/+^* and *Stim1^I115F/+^* mice, carrying heterozygous missense mutations affecting the luminal Ca^2+^-binding EF hands, primarily exhibit reduced muscle force and thrombocytopenia [19,20,21], while *Stim1^R304W/+^* mice, harboring a mutation in the cytosolic coiled-coil domain, show a multi-systemic phenotype encompassing short stature, muscle weakness, prolonged bleeding times, and spleen anomalies [22,23].

In contrast, only a single ORAI1 mouse model for TAM/STRMK exists [24]. The *Orai1^G100S/+^* mice showed reduced muscle force production together with increased serum creatine kinase (CK) levels and the appearance of tubular aggregates. However, other typical TAM/STRMK signs were not noted, merely allowing a partial insight into the common and diverging physiopathology of the different TAM/STRMK forms. The absence of an ORAI1 model with multi-systemic disease signs also impedes the development of a common therapy applicable to all TAM/STRMK patients independently of the mutation and the implicated gene. To overcome this limitation, we generated and phenotypically characterized a TAM/STRMK mouse carrying another ORAI1 GoF mutation. *Orai1^V109M/+^* mice were smaller than WT littermates and manifested muscle weakness associated with elevated basal Ca^2+^ levels and the presence of tubular aggregates in myofibers, as well as splenomegaly and thrombocytopenia.

Overall, this study contributes to a deeper understanding of the pathophysiological effect of ORAI1 GoF mutations on different tissues and organs and enables the comparison of STIM1- and ORAI1-related TAM/STRMK. It also provides the opportunity to assess therapeutic strategies for TAM/STRMK and potentially for other common or rare Ca^2+^-related disorders impacting muscle, spleen, and platelets.

## 2. Materials and Methods

### 2.1. Generation of the Orai1^V109M/+^ Mouse Model

Mice were housed in ventilated cages in temperature-controlled rooms with 12 h day light/dark cycles and free access to water and food. Animal experimentation was approved by the institutional ethics committee in accordance with French and European legislation (project #40514-2022120214489280).

The *Orai1^V109M/+^* mouse line was established at the ICS (Institut Clinique de la Souris; http://www.ics-mci.fr/en/, accessed on 2 August 2 2024) using the CRISPR/Cas9 technology. Briefly, C57BL/6N mouse embryonic stem (ES) cells were electroporated with a targeting vector carrying the GTC>ATG codon modification at cDNA positions 325–327 (NM_175423.3) with a novel *Tse*I restriction site and an auto-excision neomycin cassette. The selected clone was micro-injected into BALB/C blastocysts, and resulting male chimeras were bred with WT C57BL/6N females to obtain a founder mouse with germline transmission. The following genotyping primers were used: TTTGGCATTCCCAGAAATTGAGACTG (forward) and GGGTGACTCTTTGACCGAGTTGAGG (reverse). *Tse*I restriction results in a single amplicon of 448 bp for WT animals and in two additional amplicons of 277 and 171 bp for *Orai1^V109M/+^* mice. Kyphosis was evaluated by the angle between ears, spine, and sacrum. An angle of 130–150° was defined as low-degree kyphosis and an angle below 130° as high-degree kyphosis. With exception of the follow-up of postnatal weight gain and Ca^2+^ measurements in myoblasts, all experiments were carried out on 4-month-old mice with comparable numbers of males and females.

### 2.2. Blood Counts and Chemistry

Blood was sampled by retro-orbital puncture of the animals. Blood counts were performed on the ADVIA 120 system (Siemens, Munich, Germany) to quantify platelets. Ca^2+^ and creatine kinase (CK) levels were determined using the OLYMPUS AU-400 automated laboratory work station (Beckmann Coulter, Brea, CA, USA).

### 2.3. Muscle Contraction

Mice were anesthetized by subsequent intraperitoneal injections of domitor/fentanyl (2/0.28 mg/Kg), diazepam (8 mg/Kg), and fentanyl (0.28 mg/Kg), and the distal tendon of the tibialis anterior (TA) was excised and attached to the Complete1300A Mouse Test System (Aurora Scientific, Aurora, ON, Canada). Maximal force was assessed through electrical 1–150 Hz stimulations of the sciatic nerve or the muscle every 30 s, and specific muscle force was obtained by the division of maximal muscle force (mN) with wet muscle weight (mg). Fatigue, reflecting muscle force decrease over time, was assessed by 80 stimulations of 40 Hz with a duration of 1 s and a rest interval of 3 s.

### 2.4. Muscle and Spleen Morphology

Tibialis anterior (TA) muscle sections (8 µm) underwent hematoxylin and eosin (H&E) and Alizarin red staining for histological investigations on fiber size, nuclear positioning and intracellular Ca^2+^ load. Myofibers were delimited with the Cellpose segmentation algorithm [25], and the myofiber diameter (MinFeret) was calculated with ImageJ (version 1.54f). Images were recorded with the Nanozoomer 2HT slide scanner (Hamamatsu, Japan) and analyzed using a home-made ImageJ plugin. Semithin sections (0.5 µm) were stained with toluidine blue and viewed on a DM4000 B microscope (Leica, Wetzlar, Germany). For electron microscopy, muscle samples were fixed (glutaraldehyde 2.5% and paraformaldehyde 2% in 0.1 M cacodylate buffer, pH 7.4), post-fixed (osmium tetroxide 1% reduced by 0.8% potassium ferricyanide), incubated in 1% uranyl acetate, gradually dehydrated in ethanol, and embedded in epon 812 resin. Grids were viewed on a Hitachi H7500 transmission electron microscope (K.K. Hitachi Seisakusho, Tokyo, Japan) equipped with an AMT Hamamatsu digital camera.

Spleen was fixed in 4% paraformaldehyde and embedded in paraffin, and 5 µm sections were stained with H&E to assess histological anomalies and quantify megakaryocyte numbers with the ImageJ Cell Counter plugin.

### 2.5. Protein Level

To quantify ORAI1 protein levels, muscle samples were lysed in radio immunoprecipitation (RIPA) buffer supplemented with 1 mM PMSF, 1 mM DTT, and EDTA-free protease inhibitor cocktail (Roche, Basel, Switzerland). Protein concentrations were determined with the DC^TM^ Protein Assay kit (Bio-Rad laboratories, Hercules, CA, USA), and 10 µg of denatured protein was loaded on a 10% SDS-PAGE gel and transferred to a nitrocellulose membrane using the Transblot^®^ TurboTM RTA Transfer Kit (Bio-Rad laboratories). Membranes were blocked in Tris-buffered saline (TBS) buffer containing 5% non-fat dry milk and 0.1% Tween 20. The following primary and secondary antibodies were used: mouse anti-ORAI1 (#sc-377281, Santa Cruz Biotechnology, Dallas, TX, USA) and peroxidase-coupled goat anti-mouse (#15-036-068, Jackson ImmunoResearch, West Grove, PA, USA). Images were recorded with the Amersham Imager 600 (Amersham, UK) and the DMRXA2 microscope (Leica). Ponceau S staining (Sigma-Aldrich, St. Louis, MO, USA) served as loading control.

### 2.6. Resting Ca^2+^ Levels

Primary myoblasts from 5-day-old mice were collected as previously described [26]. Cells were plated in Iscove’s Modified Dulbecco’s Medium (IMDM) supplemented with 20% FCS, 0.1% gentamycin, and 1% chicken embryo extract (CEE) on Matrigel-coated plates (Corning Life Sciences, Corning, NY, USA) and then transferred to 35 mm dishes with 20 mm bottom well #1.5H cover glass (Cellvis, Mountain View, CA, USA) coated with Matrigel until confluency of 50–80%.

Resting cytosolic Ca^2+^ levels were quantified in myoblasts incubated with 3 µM Fura-8 AM (AAT Bioquest, Pleasanton, CA, USA), washed, and incubated in balanced salt solution (BSS) with 2 mM Ca^2+^. Ratiometric imaging was performed on a confocal TCS SP8-UV inverted microscope (Leica), and images were sequentially acquired using a 355 nm OPSL laser and a 405 nm laser diode for excitation and an HyD detector set between 475 and 600 nm. Signal ratio between fluorescence emitted following excitation at 355 nm and fluorescence emitted following excitation at 405 nm were quantified using a custom macro program developed with FiJi software (version 1.57f). In total, 3 different experiments with 4 samples per group were analyzed.

### 2.7. Statistics

Data were verified for normal distribution using the Shapiro–Wilk test and are presented as mean ± SEM. For normally distributed data, we used the Student’s *t*-test; otherwise, the Mann–Whitney U test was used. The chi-square test was used for birth ratio. Significant differences are indicated as *^/#^ *p* < 0.05, **^/##^ *p* < 0.01, ***^/###^ *p* < 0.001, and ****^/####^ *p* < 0.0001.

## 3. Results

**Figure 2:**
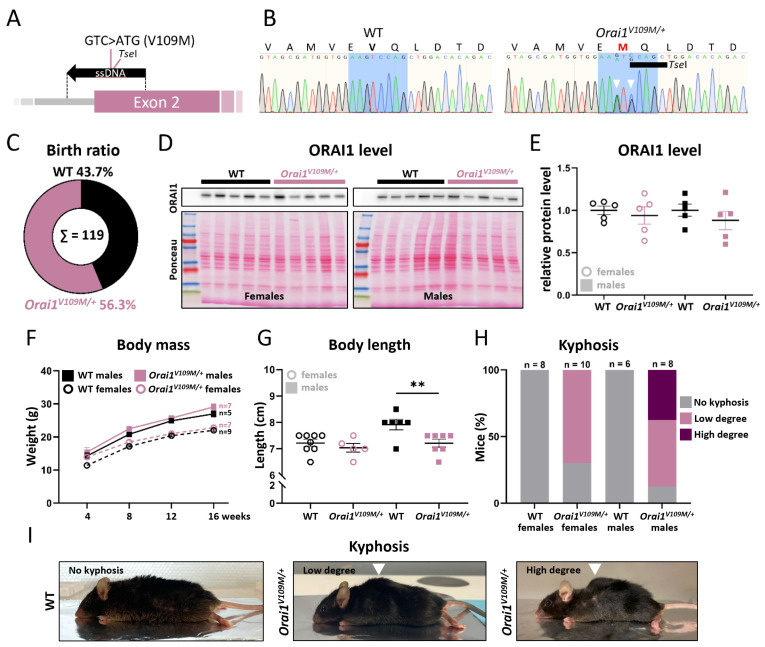
Generation of *Orai1^V109M/+^* mice and postnatal follow-up. (**A**) The ORAI1 V109M mutation was introduced into murine C57BL/6N ES cells using a single-strand DNA (ssDNA) and CRISPR/Cas9 technology. (**B**) Sanger sequencing confirmed the heterozygous GTC>ATG codon change (arrowheads) and the introduction of a *Tse*I restriction site (GCWGC) in *Orai1* exon 2. (**C**) *Orai1^V109M/+^* mice were born with Mendelian ratio. (**D**,**E**) Western blot on muscle samples and quantification of signal intensities revealed similar ORAI1 protein levels in WT and *Orai1^V109M/+^* mice. (**F**) Postnatal weight gain was comparable in WT and *Orai1^V109M/+^* mice from 4 to 16 weeks. (**G**) At 4 months, *Orai1^V109M/+^* males were smaller than WT littermates. Significant differences are indicated as ** *p* < 0.01. (**H**) Moderate or severe kyphosis was noted in the majority of *Orai1^V109M/+^* mice at 4 months and was absent in WT littermates. (**I**) Representative images showing kyphosis (arrowheads) in *Orai1^V109M/+^* males.

To complement the existing murine STIM1 and ORAI1 models for TAM/STRMK and to investigate the multi-systemic impact of ORAI1 GoF mutations, we generated *Orai1^V109M/+^* mice (corresponding to V107M in patients [15]) by introducing the GTC>ATG substitution in *Orai1* exon 2 through homologous recombination (Figure 2A,B). In contrast to *Stim1^R304W/+^* mice [23], *Orai1^V109M/+^* offspring—issued from crossings of WT males with *Orai1^V109M/+^* females or *Orai1^V109M/+^* males with WT females—were born with the expected Mendelian ratio (Figure 2C), indicating that only specific TAM/STRMK mutations increase the risk of embryonic or perinatal lethality. The ORAI1 V109M mutation did not compromise protein expression or stability as illustrated by the comparable ORAI1 levels in muscle extracts from *Orai1^V109M/+^* and WT littermates (Figure 2D,E).

*Orai1^V109M/+^* and WT mice underwent comparative phenotyping of postnatal growth, spleen histology, platelet number, and muscle function and structure to conclude on the concordance of the murine model with the human disorder and its suitability for the development of therapeutic approaches.

### 3.1. Reduced Body Length in Orai1^V109M/+^ Males and Occurrence of Kyphosis

Unlike *Stim1^R304W/+^* and *Stim1^I115F/+^* mice [20,23], *Orai1^V109M/+^* offspring showed a normal weight gain over the first 16 weeks of life (Figure 2F). However, at 4 months of age, *Orai1^V109M/+^* males had a reduced body length with an average of 7.24 cm compared with 8 cm for WT littermates, while *Orai1^V109M/+^* females were normally sized (7.21 cm versus 7.35 cm for WT females) (Figure 2G). Remarkably, the *Orai1^V109M/+^* genotype blurred the size difference but not the weight disparity between male and female mice at 4 months.

Physical examination of the animals revealed kyphosis in the majority of all *Orai1^V109M/+^* males and females. Spine curvature was more pronounced in *Orai1^V109M/+^* males with 37.5% of the animals showing high-degree kyphosis (absent in *Orai1^V109M/+^* females) and 50% showing low-degree kyphosis (70% in *Orai1^V109M/+^* females) (Figure 2H,I). Although kyphosis may partly explain the reduced body length in *Orai1^V109M/+^* males, we did not observe a clear correlation between body size and the degree of spine curvature in individual mice (*p* = 0.2). Kyphosis was not reported in other TAM/STRMK mice but is a common feature in myopathy mouse models and generally results from paraspinal and respiratory muscle weakness [27].

### 3.2. Abnormal Spleen Histology and Decreased Platelets in Orai1^V109M/+^ Mice

**Figure 3:**
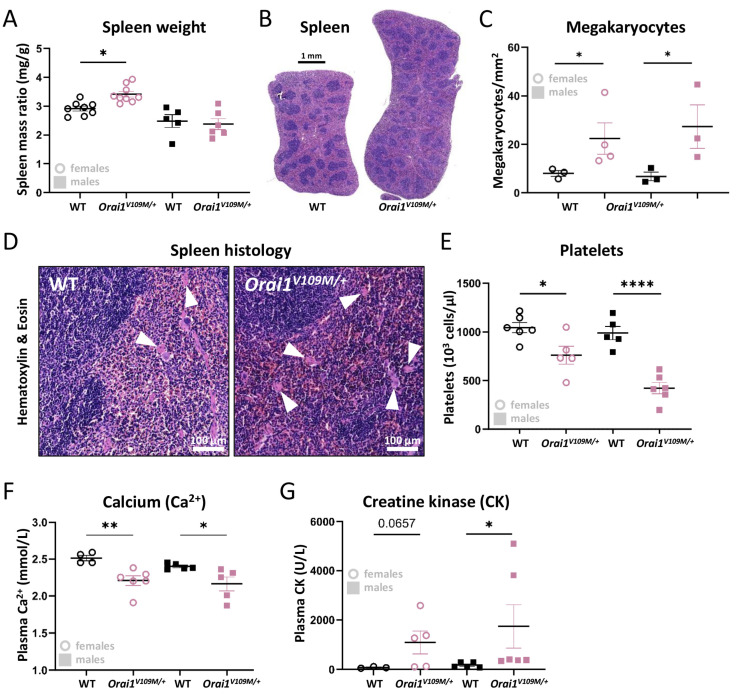
Spleen anomalies and abnormal blood parameters in *Orai1^V109M/+^* mice. (**A**) *Orai1^V109M/+^* female mice exhibited a higher spleen/body mass ratio compared with female WT littermates. (**B**) Representative images illustrating spleen enlargement in an *Orai1^V109M/+^* female mouse. (**C**) Megakaryocyte numbers were increased in *Orai1^V109M/+^* spleen from males and females compared with WT controls. (**D**) Representative images of WT and *Orai1^V109M/+^* spleen sections; megakaryocytes are indicated by arrowheads. (**E**) Compared with WT controls, platelet numbers were significantly decreased in *Orai1^V109M/+^* mice. (**F**,**G**) Biochemical blood analyses revealed hypocalcemia and elevated serum creatine kinase (CK) levels in *Orai1^V109M/+^* males, while *Orai1^V109M/+^* females showed a tendency of CK elevation. Significant differences are indicated as * *p* < 0.05, ** *p* < 0.01, and **** *p* < 0.0001.

Spleen anomalies are typical features in TAM/STRMK patients and STIM1 mice but were not noted in the existing *Orai1^G98S/+^* mouse model [7,10,11,12,16,17,20,21,23,24,28,29,30,31,32,33]. An increase in spleen weight by 17% was observed in *Orai1^V109M/+^* females, while spleens from *Orai1^V109M/+^* males were indistinguishable from the WT (Figure 3A,B). However, histological analyses of spleen sections revealed megakaryocyte hyperplasia associated with abnormal megakaryocyte distribution in both *Orai1^V109M/+^* males and females compared with WT littermates (Figure 3C,D). Megakaryocytes produce and release thrombocytes into the bloodstream, where they form hemostatic plugs at sites of vascular injury [34]. In TAM/STRMK patients and murine STIM1 models, thrombocytopenia is concomitant with prolonged bleeding times [7,10,11,15,16,17,20,21,23,28,29,30,31,32,33,35,36], and accordingly, *Orai1^V109M/+^* mice displayed a reduction in circulating platelets (Figure 3E). Biochemical tests on *Orai1^V109M/+^* blood samples also revealed hypocalcemia (Figure 3F), commonly observed in TAM/STRMK patients [6,11,15,16,17,31,33,37], as well as a tendency of elevated creatine kinase levels (CK, Figure 3G), a typical feature of TAM/STRMK and other muscle disorders involving myofiber degeneration [9,12,38].

### 3.3. Reduced Muscle Force and Elevated Basal Ca^2+^ Levels in Orai1^V109M/+^ Mice

**Figure 4:**
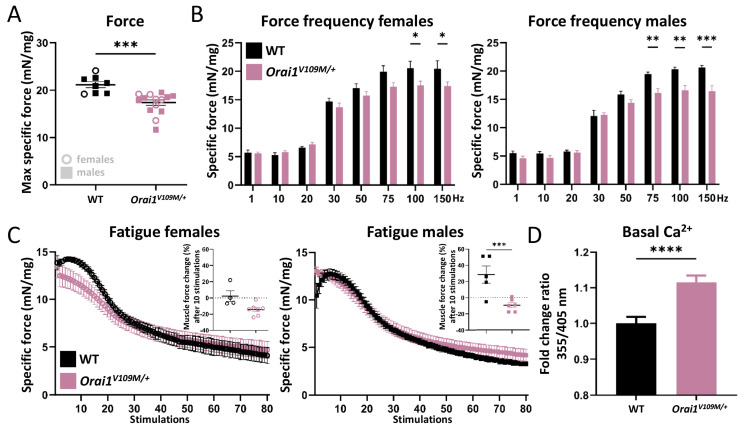
Abnormal muscle function in *Orai1^V109M/+^* mice. (**A**) Force transduction experiments disclosed reduced maximal force of *Orai1^V109M/+^* tibialis anterior compared with WT. (**B**) Increasing stimulation frequencies evidenced normal submaximal force in *Orai1^V109M/+^* mice but reduced maximal force compared with WT littermates. (**C**) *Orai1^V109M/+^* mice showed abnormal fatigue curves with absence of muscle force increase in *Orai1^V109M/+^* males after 10 stimulations and a similar tendency in *Orai1^V109M/+^* females (insets). (**D**) Ratiometric analyses revealed increased resting Ca^2+^ levels in isolated *Orai1^V109M/+^* myoblasts compared with WT controls. Significant differences are indicated as * *p* < 0.05, ** *p* < 0.01, *** *p* < 0.001, and **** *p* < 0.0001.

In situ muscle force and resistance to fatigue was quantified on anesthetized animals following electrical stimulation of the tibialis anterior. Both *Orai1^V109M/+^* male and female mice manifested reduced maximal force compared with WT littermates (Figure 4A). Further analysis of muscle contractility revealed that muscle force of *Orai1^V109M/+^* mice was normal at low stimulation frequencies of 20–50 Hz and decreased at higher stimulation frequencies of 75–150 Hz compared with WT littermates (Figure 4B). This is similar to *Orai1^G98S/+^* mice [24] and partly different from *Stim1^R304W/+^* mice, which exhibited premature muscle contraction at low stimulation frequencies and reduced maximal force at high stimulation frequencies [23]. Moreover, we determined muscle force decrease after repetitive stimulations, and we found that the fatigue curve of *Orai1^V109M/+^* mice diverged from the shape of the WT control (Figure 4C) and was comparable with fatigue curves from *Stim1^R304W/+^* mice [23].

To further investigate muscle physiology and the causes of abnormal muscle contraction, we isolated primary myoblasts from WT and *Orai1^V109M/+^* mice. Ratiometric analyses disclosed a significant increase in resting Ca^2+^ levels in *Orai1^V109M/+^* myoblasts compared with WT controls (Figure 4D). Elevated cytosolic Ca^2+^ concentrations or increased extracellular Ca^2+^ entry was also observed in myotubes or myofibers from STIM1 and ORAI1 mice [19,20,23,24], as well as in myoblasts, fibroblasts, lymphocytes, or platelets from TAM/STRMK patients [5,6,7,10,11,18,39], and were shown to correlate with impaired muscle contraction and relaxation kinetics [40].

### 3.4. Myofiber Atrophy and Tubular Aggregates in Orai1^V109M/+^ Mice

**Figure 5:**
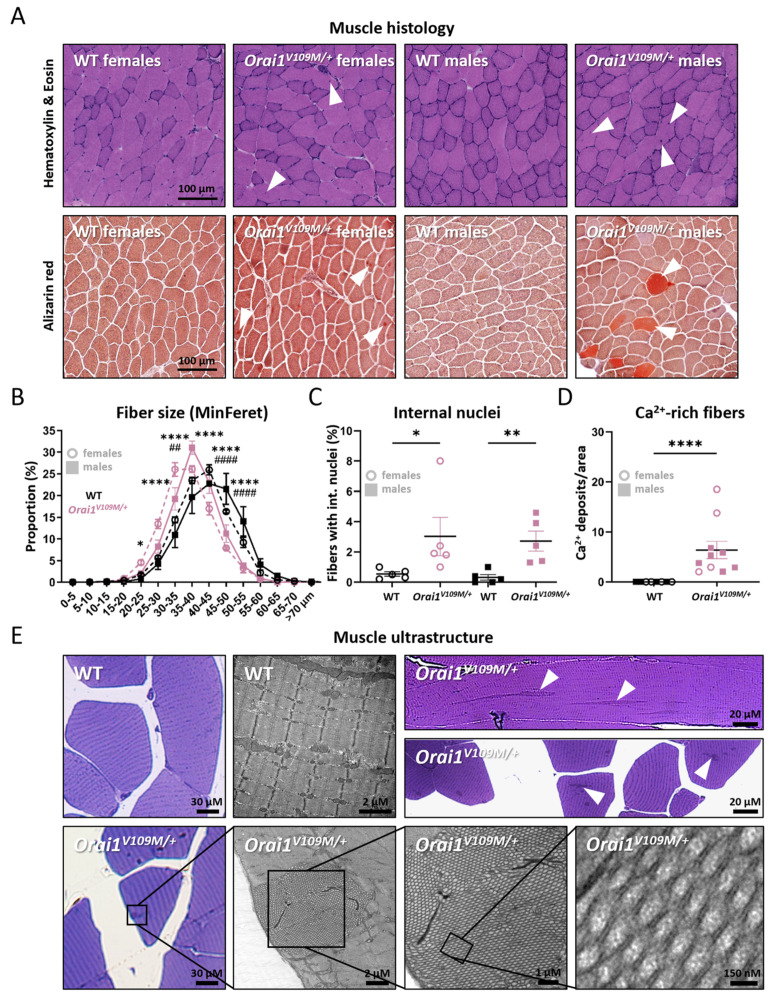
Abnormal muscle structure in *Orai1^V109M/+^* mice. (**A**) Representative images of transverse sections of WT and *Orai1^V109M/+^* tibialis anterior stained with hematoxylin and eosin and Alizarin red. Arrowheads indicate internal nuclei and Ca^2+^ deposits/Ca^2+^-rich myofibers. (**B**,**C**) *Orai1^V109M/+^* mice exhibited a higher ratio of smaller muscle fibers and internal nuclei compared with WT littermates. (**D**) *Orai1^V109M/+^* muscle sections showed a significantly higher ratio of Ca^2+^-rich fibers compared with controls. (**E**) Toluidine blue-stained semithin sections and electron microscopy evidenced the presence of tubular aggregates (arrowheads) in longitudinal and transversal *Orai1^V109M/+^* muscle sections (**top right**), while no tubular aggregates were detected in WT animals (**top left**). The lower panel shows tubular aggregates on semithin and EM sections from *Orai1^V109M/+^* mice with increasing magnification. Significant differences are indicated as * *p* < 0.05, **^/##^ *p* < 0.01, and ****^/####^ *p* < 0.0001.

To correlate muscle function with muscle structure, tibialis anterior sections from *Orai1^V109M/+^* mice and healthy controls underwent morphological analyses by light and electron microscopy. Histological examination of transverse sections revealed a decrease of average fiber diameter and an increased ratio of internalized nuclei and Ca^2+^-rich fibers in *Orai1^V109M/+^* mice, all indicating enhanced muscle fiber degeneration (Figure 5A–D). In general, Ca^2+^ deposits were more prominent in *Orai1^V109M/+^* males compared with *Orai1^V109M/+^* females. Moreover, correlated light and electron microscopy (CLEM), combining toluidine blue-stained semithin sections with ultrastructural investigations, uncovered the presence of tubular aggregates (Figure 5E), constituting the principal histopathological hallmark in biopsies from TAM/STRMK patients [1,41]. This is of particular interest since tubular aggregates were also observed in *Orai1^G98S/+^* mice [24] but were undetectable in all murine STIM1 models for TAM/STRMK [19,20,21,22,23]. With a length of up to 75 µM, a width ranging from 2 to 8 µM, and a cross-section diameter of individual membrane tubules of 80–90 nm, the tubular aggregates detected in *Orai1^V109M/+^* male and female mice were of comparable aspect and size as those described in TAM/STRMK patients [1].

## 4. Discussion

Here, we describe the generation and characterization of a novel mouse model for tubular aggregate myopathy (TAM) and Stormorken syndrome (STRMK), two clinically overlapping disorders affecting skeletal muscle, spleen, and platelets. The *Orai1^V109M/+^* mice showed muscle weakness associated with myofiber atrophy, internalized nuclei, elevated resting Ca^2+^ levels, and abundance of tubular aggregates, as well as short stature, splenomegaly, and thrombocytopenia.

The V109M missense mutation (corresponding to V107M in TAM/STRMK patients [15]) affects the pore-forming transmembrane domain of ORAI1 and resides in direct proximity to the glutamic acid residue E106, which confers high selectivity for Ca^2+^ ions [42]. Functional tests in cell models demonstrated a dual pathogenic effect of the mutation on both channel permeability and Ca^2+^ selectivity, resulting in excessive extracellular entry of Ca^2+^ and other cations [15,43].

### 4.1. Orai1^V109M/+^ Mice Recapitulate Main Signs of the Human Disorder

The majority of all reported TAM/STRMK patients carry mutations in the Ca^2+^ sensor STIM1. ORAI1 mutations are less frequent, and to date, only eight families have been described [6,7,15,18]. The clinical spectrum of affected individuals ranges from marked childhood-onset muscle weakness, joint contractures, rigid spine, miosis, ichthyosis, and bleeding episodes to mild adulthood-onset muscle weakness and cramps, and the phenotypic severity correlates with the position of the ORAI1 mutation [15,18].

In general, patients with ORAI1 mutations primarily present with a skeletal muscle phenotype, while multi-systemic features are less prevalent in comparison with STIM patients [12]. Indeed, miosis has only been reported in four ORAI1 families [7,15,18], hypocalcemia in three families [6,15], dyslexia or intellectual disability in two families [6,15], and ichthyosis and bleeding diathesis in a single family [15]. Short stature and spleen anomalies, commonly observed in STIM1 patients, have not been described in ORAI1 patients and may reflect physiopathological differences between both TAM/STRMK forms. Alternatively, both phenotypes might represent rare, mild, or gender-specific features which escaped detection in the few reported ORAI1 families. This is supported by the occurrence of moderate splenomegaly only in *Orai1^V109M/+^* female mice and reduced body length only in *Orai1^V109M/+^* male mice. *Orai1^V109M/+^* mice also exhibited moderate hypocalcemia and thrombocytopenia, which were only reported in a subset of ORAI1 patients. Blood counts and biochemistry may not have been examined in other affected individuals or were possibly within normal ranges. Importantly, *Orai1^V109M/+^* mice form a genetically homogenous cohort and were analyzed in statistically significant numbers, which facilitates the detection of small disparities. This is different from the few and genetically heterogeneous ORAI1 patients of diverging geographic and ethnic origin.

Overall, the *Orai1^V109M/+^* mice described in the present study manifested muscle weakness in combination with moderate multi-systemic signs, which is in accordance with the clinical picture of ORAI1 patients. These findings highlight and define the primary defects caused by ORAI1 GoF mutations, whereas the genetic background and the modulating activity of modifier genes possibly account for additional clinical findings in single TAM/STRMK patients with ORAI1 mutations.

### 4.2. Common Features and Differences Between Murine TAM/STRMK Models

To date, five murine TAM/STRMK models have been described [20,21,22,23,24]. All carry STIM1 or ORAI1 gain-of-function mutations and differ in phenotypic severity and the presence or absence of multi-systemic signs. While *Orai1^G98S/+^*, *Stim1^D84G/+^* and *Stim1^I115F/+^* mice essentially manifest muscle weakness with or without prolonged bleeding times [19,20,21], only *Stim1^R304W/+^* mice showed the full TAM/STRMK phenotype with additional incidence of short stature, hypocalcemia, eye movement defects, and skin and spleen anomalies [23].

Transcriptomics on muscle samples from *Stim1^R304W/+^* mice disclosed aberrant expression profiles of genes implicated in excitation–contraction coupling (ECC) and Ca^2+^ handling, resulting in abnormal muscle contraction kinetics and reticular stress and ultimately leading to enhanced myofiber degeneration, mitochondrial loss, and muscle weakness [40]. The higher resting Ca^2+^ levels in human TAM/STRMK platelets were shown to induce a pre-activation state impacting on thrombocyte morphology [30], and analysis of the coagulation defects in *Stim1^D84G/+^* mice revealed a higher clearance rate of the platelets [21].

A common feature of all TAM/STRMK mouse models with STIM1 mutations is the absence of tubular aggregates on muscle sections. This contrasts the findings in *Orai1^G98S/+^*/*Orai1^V109M/+^* mice and muscle biopsies from TAM/STRMK patients with CASQ1 [2,3], STIM1 [5,7,10,11,44], ORAI1 [6,15], or RyR1 [4] mutations, all invariably showing eponymous tubular aggregates as a typical histopathological feature. Nevertheless, murine STIM1 models exhibit a muscle phenotype, suggesting that muscle weakness and the occurrence of tubular aggregates are not causally linked and indicating that muscle weakness rather arises from a combination of impaired muscle contraction and relaxation kinetics, myofiber degeneration, and cellular Ca^2+^ stress.

Tubular aggregates contain large amounts of Ca^2+^ and sarcoplasmic reticulum (SR) proteins such as STIM1, CASQ1, RyR1, or SERCA1/2, and are therefore believed to originate from the SR [41]. The precise way of tubular aggregate formation remains to be elucidated, but it is conceivable that the abundance of Ca^2+^ in the SR triggers protein misfolding and aggregation, leading to SR dilatation and ultimately to the appearance of membrane stacks as precursors of tubular aggregates [12]. It is also possible that the tubular aggregates exert a protective role in *Orai1^G98S/+^*/*Orai1^V109M/+^* mice and human TAM/STRMK muscle by trapping misfolded proteins and excessive Ca^2+^ to reduce cellular stress and prevent myofiber breakdown [45]. Consistently, *Stim1^D84G/+^*, *Stim1^I115F/+^*, and *Stim1^R304W/+^* mice display significantly more dystrophic signs of muscle fiber degeneration including nuclear internalization, fiber atrophy, and infiltrations of immune cells compared with *Orai1^G98S/+^*/*Orai1^V109M/+^* mice and TAM/STRMK patients.

## 5. Concluding Remarks

*Orai1^V109M/+^* mice faithfully recapitulate the main signs of the human disorder, indicate the primary defects caused by ORAI1 GoF mutations, and complement the currently available STIM1 and ORAI1 mouse models. They represent valuable tools to investigate and compare the cellular pathways and processes implicated in disease development, examine the formation of tubular aggregates and their physiopathological effect, and can serve for the establishment and validation of treatments.

This is of particular importance in view of the recent therapeutic advances in preclinical models. The decrease of *Orai1* expression or ORAI1 permeability efficiently anticipated disease development in *Stim1^R304W/+^* mice [46,47], and the SOCE inhibitor CIC-39 partially resolved the phenotype in *Stim1^I115F/+^* mice [48]. These findings highlight ORAI1 as a main target for a common therapy for all TAM/STRMK forms and show that small molecules able to reduce extracellular Ca^2+^ entry represent promising therapeutic strategies. It is worth mentioning that several ORAI1 inhibitors have been developed and tested in diverse disorders including asthma, COVID-19 pneumonia, refractory lymphomas, and acute pancreatitis [49,50,51]. A positive outcome of these clinical trials may represent additional and alternative treatment options for TAM/STRMK and other Ca^2+^-related diseases.

## Data Availability

The authors confirm that the data supporting the findings of this study are available within the article.
